# Intranasal Administration of PACAP Is an Efficient Delivery Route to Reduce Infarct Volume and Promote Functional Recovery After Transient and Permanent Middle Cerebral Artery Occlusion

**DOI:** 10.3389/fendo.2020.585082

**Published:** 2021-01-20

**Authors:** Asma Cherait, Julie Maucotel, Benjamin Lefranc, Jérôme Leprince, David Vaudry

**Affiliations:** ^1^ Normandie Univ, UNIROUEN, Inserm U1239, Laboratory of Neuronal and Neuroendocrine Communication and Differentiation, Neuropeptides, Neuronal Death and Cell Plasticity Team, Rouen, France; ^2^ Department of Natural and Life Sciences, Faculty of Sciences, University of Algiers, Algiers, Algeria; ^3^ Laboratory of Valorization and Bioengineering of Natural Resources, University of Algiers, Algiers, Algeria; ^4^ Normandie Univ, UNIROUEN, Regional Cell Imaging Platform of Normandy (PRIMACEN), Rouen, France

**Keywords:** pituitary adenylate cyclase-activating polypeptide, intranasal administration, cerebral ischemia, infarct volume, functional recovery

## Abstract

Intranasal (IN) administration appears to be a suitable route for clinical use as it allows direct delivery of bioactive molecules to the central nervous system, reducing systemic exposure and sides effects. Nevertheless, only some molecules can be transported to the brain from the nasal cavity. This led us to compare the efficiency of an IN, intravenous (IV), and intraperitoneal (IP) administration of pituitary adenylate cyclase-activating polypeptide (PACAP) after transient or permanent middle cerebral artery occlusion (MCAO) in C57BL/6 mice. The results show that the neuroprotective effect of PACAP is much more efficient after IN administration than IV injection while IP injection had no effect. IN administration of PACAP reduced the infarct volume when injected within 6 h after the reperfusion and improved functional recovery up to at least 1 week after the ischemia.

## Introduction

Stroke can either be hemorrhagic, when a cerebral blood vessel bursts, or ischemic, when an artery gets obstructed by a clot (1). In 85% of the cases, stroke is of ischemic origin due to a permanent or temporary occlusion of a cerebral artery, which in one third of the patients concerns the middle cerebral artery (MCA) (2). Stroke induced blood flow disruption, impairs oxygen and nutrients supply, inducing irreversible neuronal loss responsible for cognitive disorders, fatigue, paralysis, and even death of the patient. Actually, stroke is the second leading cause of death worldwide and the third leading cause of disability in adults (3). Stroke affects almost 17 million people worldwide each year, i.e., one victim every 2 s (4). As a consequence, millions of stroke survivors must adapt to a life with restrictions in daily activities and depend on other’s continuous support (5).

A prolonged and severe reduction of cerebral blood flow (CBF) causes neuronal necrosis responsible for irreversible neurological deficits. In contrast, a moderate and gradual decline in CBF at a distance from the occlusion induces a benign oligemia which can be tolerated and remains asymptomatic. In between the core where necrosis occurs and the peripheral benign oligemia, the ischemic penumbra is a territory where neurons are on the verge of death but are still salvageable by rapid blood flow restoration and/or proper pharmacological treatment (6).

The approved medical treatments for acute ischemic stroke are intravenous (IV) thrombolysis with recombinant tissue plasminogen activator (tPA) and/or mechanical thrombectomy, which promote the recanalization of the occluded artery and therefore the rapid restoration of a normal CBF. However, tPA can only be administered within the first 4.5 h after the manifestation of the symptoms since after this short therapeutic time window tPA administration becomes deleterious (7). Mechanical thrombectomy can be carried out in people with large vessel ischemic stroke within the first 6 h after the occlusion but needs competent practitioners for this act and access to an appropriate diagnostic imaging unit (8). Application of these very limited therapeutical options is also restricted by cost, time and age of patients, so that less than 15% of stroke victims benefit from thrombolysis. Furthermore, functional recovery after thrombolysis is only partial but so far, all other therapeutic approaches targeting the acute phase of stroke have failed (9, 10). Thus, there is an urgent need for additional, more widely applicable, treatment options.

Pituitary adenylate cyclase-activating polypeptide (PACAP) is a neuropeptide of the VIP/secretin/glucagon/growth hormone-releasing hormone family, naturally produced by the organism and widely distributed in the brain and peripheral tissues, notably in the endocrine pancreas, gonads, respiratory and urogenital tracts (11). PACAP acts as a neurohormone, neurotrophic factor and neuroprotective agent, through three receptors, i.e., PAC1, VPAC1, and VPAC2 (12). PACAP has high affinity for the PAC1, VPAC1, and VPAC2 receptors, whereas VIP has ~1,000 times greater affinity for VPAC receptors than for the PAC1. The 3 receptors are largely distributed in peripheral organs (13), but PAC1 is more expressed than VPAC1 and VPAC2 in the central nervous system (14). In relationship with their large distribution, activation of those three G-protein coupled receptors initiates multiple signaling pathways involved in the regulation of important biological functions such as stress response, cardiovascular effects, food intake, circadian rythm, and reproduction (15, 16).

Besides those effects on the control of physiological functions, numerous studies have highlighted the neuroprotective potential of PACAP in various neurological diseases involving neuronal cell death (17–20). For instance, PACAP protects dopaminergic neurons in a model of Parkinson’s disease (21, 22), reduces amyloidopathy in a model of Alzheimer’s disease (23–25), suppresses cortical damages in mice after traumatic brain injury (26), and improves memory performance in a model of Huntington’s disease (27).

PACAP also decreases the infarct volume and improves functional recovery after stroke. One particularity of PACAP is that it counteracts many of the deleterious processes activated by stroke through its anti-excitotoxicity, anti-apoptotic, anti-inflammatory, antioxidant, and immuno-modulatory activities (28–31). Furthermore, PACAP may act beyond the acute phase of stroke by promoting neurogenesis, plasticity and angiogenesis (32, 33).

However, to consider possible clinical applications, several parameters remain to be clarified, such as the optimal administration route, the lower efficient dose and the therapeutic window of PACAP. The present study aimed to investigate some of these issues in 2 stroke animal models.

## Material and Methods

### Animals

Two-month-old C57BL/6 (27.3 ± 2.3 g; Janvier Labs) male mice, kept in a humidity- and temperature-controlled environment under an established photoperiod with free access to food and water, were used in this study. Experiments were approved by the regional committee of ethics for animal experimentation (CENOMEXA; approval number #7619-2016101417048165) and conducted in an accredited animal facility (C7645104), according to the recommendations of the European Union under the supervision of authorized investigators.

### Reagents

PACAP38 was synthesized using solid phase strategy combined with the Fmoc chemistry methodology as previously described (34). For administration, PACAP was dissolved in a 0.9% NaCl solution.

### tMCAO and pMCAO Chirurgical Procedures

Occlusion was performed on the MCA, because approximately 70% of the human ischemic strokes affect this artery (10).

Transient middle cerebral artery occlusion (tMCAO) was induced under general anesthesia (isoflurane 1.5% to 2% infused by air) by occlusion of the right MCA by the mean of the intraluminal filament technique (35). Briefly, a nylon thread (0.1 mm in diameter) with a distal cylinder (1 mm in length and 0.18 mm in diameter) was inserted into the lumen of the internal carotid artery and advanced to the origin of the MCA. The nylon thread was removed 45 min later to allow reperfusion. To monitor the occlusion, a laser-Doppler flowmeter probe (0.7 mm in diameter, FloLab Moor Instruments) was positioned and glued on the right parietal bone, after skin and muscle incision, and then CBF was measured continuously before the occlusion and up to 10 min after reperfusion in order to monitor the efficiency of the occlusion and success of the reperfusion. Based on those laser-Doppler flowmeter recordings, 11% of the operated animals were excluded from further analysis. For sham-operated animals, the nylon thread was put in place but not pushed to the origin of the MCA. Each treated mouse received 1 h after waking up and 24 h later, 500 μl of NaCl 0.9% by subcutaneous injection to limit dehydration.

Permanent middle cerebral artery occlusion (pMCAO) was induced under general anesthesia (isoflurane 1.5% to 2% infused by air) using bipolar-forceps connected to a high frequency coagulation generator (KLS Martin ME 102). Briefly, skin was incised between the ear and the eye, the temporal muscle was detached from the skull without totally removing it and the MCA was localized below the transparent skull. The bone was carefully withdrawn above the artery. Once the MCA exposed, the artery was electrocoagulated with bipolar forceps. After checking the absence of recanalization, the temporal muscle was replaced and wound sutured. No electrocoagulation was performed on sham operated mice.

Following MCAO, mice were randomized in 3 groups, i.e., NaCl group (composed of ischemic mice treated with 0.9% NaCl), a Sham group (composed of mice having undergone all the surgical procedure but without occlusion of the MCA and treated with 0.9% NaCl) and a PACAP group (composed of ischemic mice treated with various concentrations of PACAP according to the different protocols).

Behavioral tests were performed 2 and 8 days after stroke. Brains were collected at different time points in order to perform histological experiments to measure infarct volumes and/or to quantify gene expression.

### PACAP Administration Routes

**Scheme 1:**
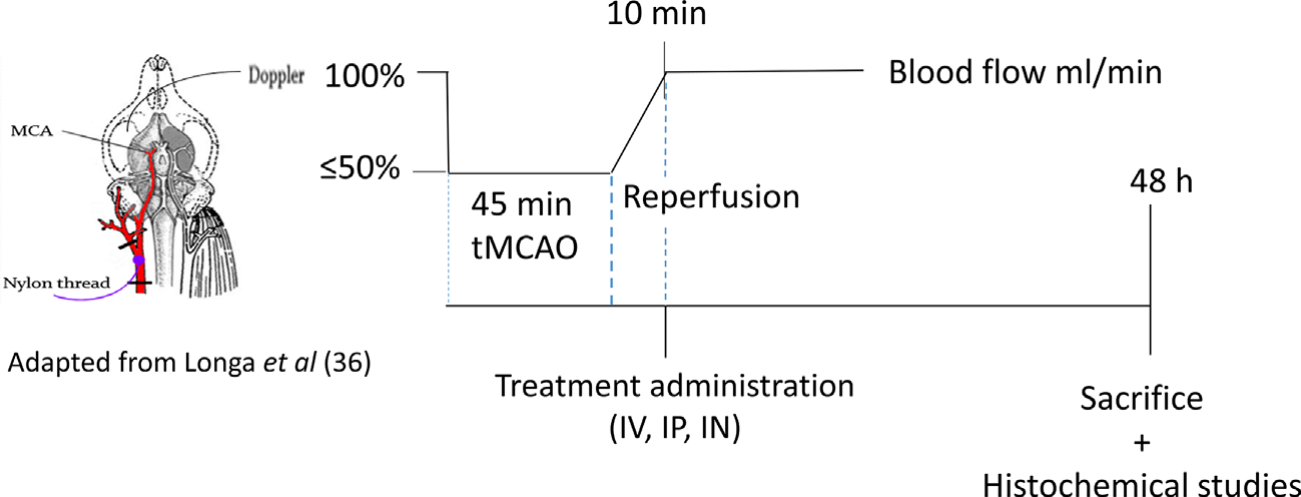
The schema of PACAP administration routes.

After tMCAO, mice were randomized into four groups corresponding to the different types of treatment: NaCl group (control), IV group, IP group, and IN group. Ten minutes after reperfusion, mice from the control group (Ctrl, n = 8) received 200 μl of NaCl intravenously; those from the IV group (IV, n = 7) received 200 μl of PACAP at the concentration of 0.02 μg/kg with 50% of the dose given as a bolus while the rest was provided as a 30-min infusion; those from the intraperitoneal (IP) group (IP, n = 8) received 200 μl of PACAP as a bolus at a concentration of 0.02 μg/kg; and finally those from the intranasal (IN) group (IN, n = 7) received 10 μl of PACAP solution at a concentration of 1 μg/μl. Forty-eight hours after reperfusion brains were collected for morphological analysis and gene expression studies (**Scheme 1**). The choice of these doses was based on the most efficient dose reported by Dejda et al. for IV injection (28) and the one used by Rat et al. for IN administration (23).

### PACAP Therapeutic Window

**Scheme 2:**
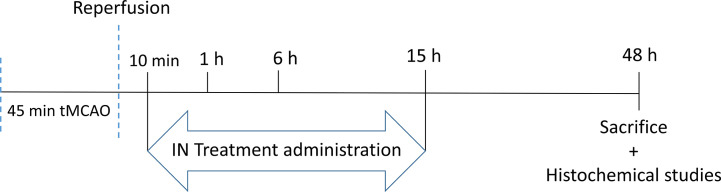
The schema of PACAP therpauetic window.

After tMCAO, mice were randomized into five groups corresponding to the different times of treatment after stroke: a NaCl group who received 10 μl of NaCl intranasally 10 min after reperfusion and 4 groups who received 10 μl of PACAP at a concentration of 1 μg/μl intranasally 10 min, 1 h, 6 h, or 15 h after reperfusion. Forty-eight hours after reperfusion brains were collected for further analysis (**Scheme 2**).

### PACAP Dose Response Efficiency

**Scheme 3:**
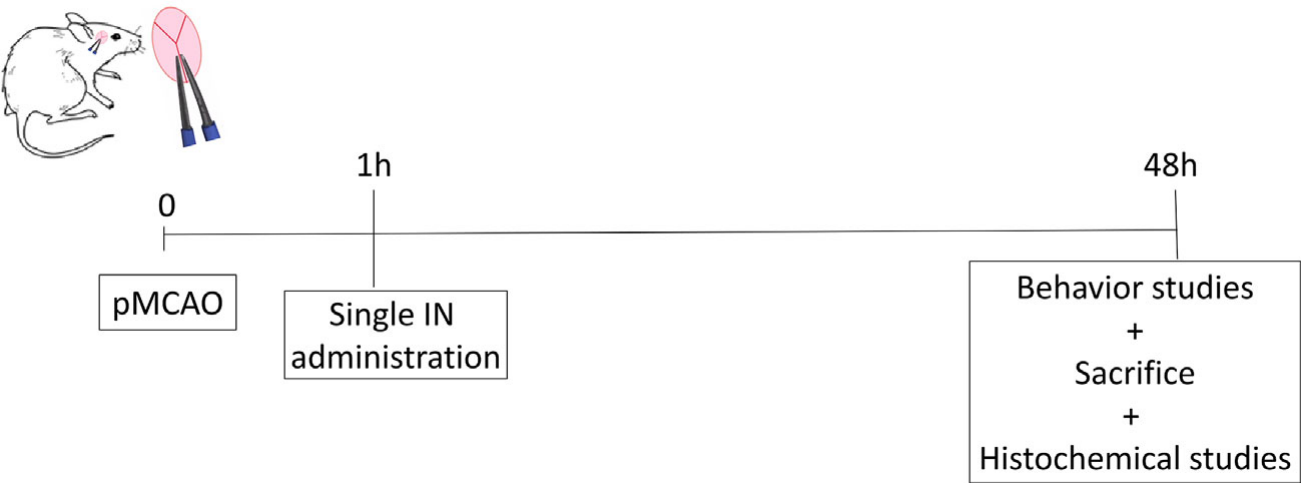
The schema of PACAP dose response efficiency.

Animals were divided into NaCl, Sham and PACAP groups. PACAP animals were treated with 4 different concentrations of peptide ranging from 1 μg/μl to 1 fg/µl. Excepted sham animals, all other mice were subjected to permanent occlusion of the middle cerebral artery (pMCAO). One hour after cerebral ischemia animals received a single IN administration of 10 µl of PACAP or Nacl. Behavioral studies were conducted 48 h after treatment, just before animals were sacrificed and brains collected (**Scheme 3**).

### PACAP Delayed IN Daily Administration Efficiency

To determine whether delayed IN delivery of PACAP durably improves functional recovery after cerebral ischemia, mice received, 6 h after pMCAO, an IN injection of a 0.9% NaCl solution (control, sham) or of a PACAP solution (1 μg/μl or 1 ng/µl). Such IN administration was repeated daily until day 6 (D6).

**Scheme 4:**
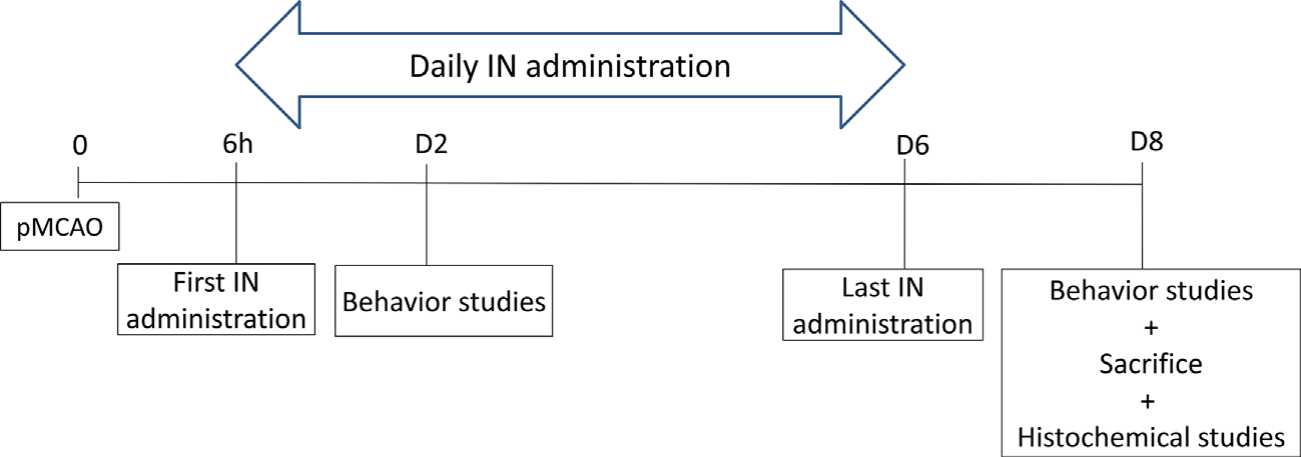
The schema of PACAP delayed IN daily administration efficiency.

Behavioral studies were performed 48 h (on day 2; D2) and 192 h (on day 8; D8) after pMCAO. The animals were sacrificed on day 8 and brains collected (**Scheme 4**).

### Functional Recovery Assessment

The **walking fault test** was used to detect deficits in motor skills and balance (36). In this test, the mouse had to walk across an elevated wooden beam (1 m long and 9 mm of diameter) to reach a safe platform (home cage). The time to cross the beam and the number of walking faults were scored. The **adhesive removal test** was used to assess sensorimotor deficits (37). Performance on this test was quantified by measuring the time taken by the mouse to detect the presence of the adhesive (first contact) and the time taken by the mouse to remove it from both paws.

### Infarct Volume Measurement

After sacrifice, brains were collected and frozen in cooled isopentane for cryosectionning. Twenty-micron thick sections were mounted on slides and stained with cresyl violet according to the Kapelsohn’s method. The slides were scanned and the measurement of infarct volume was performed by using ImageJ program. Infarct volumes were then calculated as follows:

$$v_{c}=v_{i}\left[v_{i}-\left(\frac{v_{ih}}{v_{ih}}-v_{\text{ch}}\right)\right]$$

*v_c_*, corrected volume; *v_i_*, infarct volume; *v_ih_*, ipsilateral hemisphere volume; and *v*_ch_, controlateral hemisphere volume.

### Animal Weight Monitoring and Food Intake

Animals body weight was assessed before MCAO surgery (day 0) and then daily until sacrifice. For the food intake experiments, animals have been fasted overnight. After NaCl or PACAP (10 µg/µl) IN administration, pre-weighted pellets were provided and then food consumption was recorded every 30 min for 3 h using metabolic cages.

### Gene Expression Analysis (qPCR)

Some brain tissue from ipsilateral hemispheres was collected from a series of crysotat’s brain sections (20-µm thick) in TRI-reagent (Sigma) and RNAs were extracted according to the manufacturer’s protocol. RNAs were further purified with the NucleoSpin^®^ RNA II kit (Macherey-Nagel) and total RNAs were reverse transcribed into cDNA with the Promega^®^ ImProm-II Reverse Transcription System kit. Real-time polymerase chain reaction was performed on a QuantStudio 12K Flex (Life Technologies) to quantify the expression of genes potentially regulated by ischemia.

### Statistical Analysis

Results are expressed as mean ± SEM (n ≥ 6 animals per group). We used ANOVA tests followed by *post hoc* tests for statistical analysis thanks to GraphPad Prism 6. A value of *P* * < 0.05 was considered as significant.

## Results

### PACAP Administration Route

**Figure 1:**
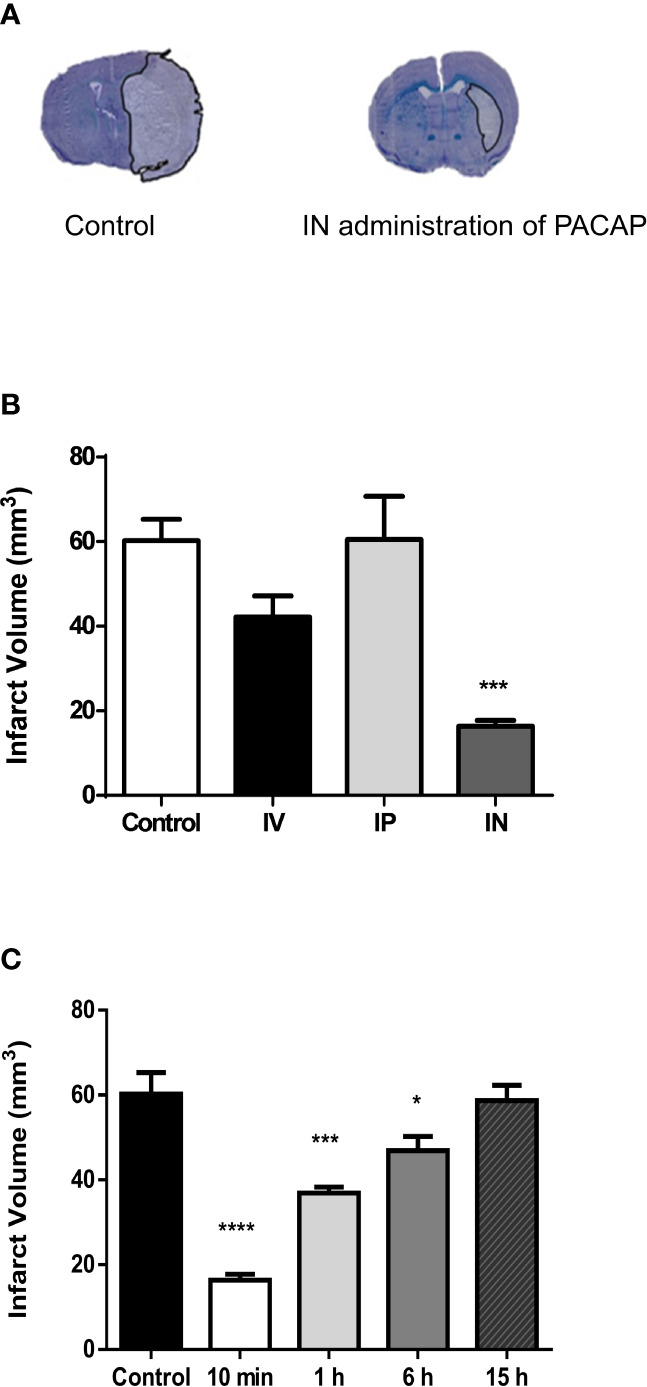
Effect on cerebral infarct volume of PACAP administration 2 days after tMCAO. **(A)** Illustration of the brain infarct area after NaCl (Control) or PACAP (IN administration of PACAP) intranasal administration, 48 h after tMCAO. **(B)** Quantification of the brain infarct volume of mice treated 10 min after the reperfusion by intranasal administration of NaCl (0.9%; Ctrl; n = 7), intravenous administration of PACAP (200 μl at a concentration of 0.02 μg/kg with 100 μl in the form of a bolus and then 100 μl by infusion over a 30-min period; IV; n = 7), intraperiotoneal administration of PACAP (200 μl in the form of a bolus at a concentration of 0.02 μg/kg; IP; n = 8) or intranasal administration of PACAP (10 µg in 10 µl; IN; n = 7), 48 h after a 45-min tMCAO. **(C)** Quantification of the brain infarct volume of mice treated 10 min (n = 8), 1 h (n = 8), 6 h (n = 8) or 15 h (n = 7) after reperfusion by an intranasal administration of PACAP (10 µg in 10 µl; IN). Control animals (Ctrl; n = 7) received 10 µl NaCl 0.9% IN administration. Volume was expressed in mm^3^. ANOVA followed by the Bonferroni’s test. *P < 0.05; ***P < 0.001; ****P < 0.0001 vs. Ctrl.

In the present study, the efficiency of IV administration, which has already been used in previous stroke studies (28–39), was compared with IP and IN administration routes. The results show that PACAP IN administration 10 min after reperfusion induced a three-fold reduction of the infarct volume (**Figure 1A**). This neuroprotective action of a PACAP IN delivery was much more efficient than the IV treatment, while the IP administration had no effect (**Figure 1B**).

### PACAP Therapeutic Window

In a second step, the neuroprotective effect of a delayed single IN PACAP administration 10 min, 1 h, 6 h and 15 h after reperfusion was investigated in a tMCAO mice model. Those experiments were conducted using a single IN administration of 10 µl of PACAP at a concentration of 1 µg/µl, which was, as indicated in **Figure 1B** the most efficient. The results show that 10 µl of PACAP at a concentration of 1 µg/µl induced 48 h after the occlusion a decrease of the lesioned volume of 72.8%, 38.7%, and 22.1% compared to NaCl treated animals when administered 10 min, 1 h, or 6 h after reperfusion, respectively (**Figure 1C**). In contrast, when IN administration of PACAP was conducted 15 h after reperfusion, the peptide had no more effect.

### Gene Expression (qPCR)

Stroke is a complex multifunctional physiopathology and MCAO activates a diversity of cellular and molecular mechanisms related to excitotoxicity, oxidative stress, post-ischemic inflammation and apoptosis (40, 41). To analyse and compare the different mechanisms potentially involved in the effects of PACAP IN administration 10 min, 1 h, 6 h, and 15 h after reperfusion in a tMCAO mice model, a transcriptomic analysis was performed by measuring the expression of 88 genes known to be regulated after stroke. Among them, 42 were indeed regulated 48 h after tMCAO in one of the experimental conditions (**Table 1**). The results indicate that 64% of these 42 genes were regulated by stroke with only 4 genes repressed, all the others being increased. Among the 27 genes regulated by stroke, 89% were also regulated by PACAP after stroke in an opposite manner, showing that the peptide exerts a strong anti-excitotoxicity, antioxidant, anti-inflammatory, and anti-apoptotic effect as evidenced by its ability to inhibit the expression of genes such as GJB6, NOS-1, IL6, BCL10, and caspase-9 (CASP9) which were induced by stroke. On the opposite, PACAP could counteract the decrease of genes such as Vegfa involved in vascular remodelling after stroke. PACAP also upregulated the brain expression of 15 genes not affected by stroke such as NEUROD1 or BDNF but known to promote synaptic plasticity and which contribute to improve functional recovery. Among the 42 genes of our list, only 4 were not regulated by PACAP, 24 being regulated when PACAP was injected 10 min after reperfusion, 18 when PACAP was injected 1 h after reperfusion, 19 when PACAP was injected 6 h after reperfusion and 17 when PACAP was injected 15 h after reperfusion. This highlights that most genes were regulated at different time points of PACAP treatment even if an early injection of PACAP after stroke affects more genes. Some genes such as CASP9, Tpa, or IL6 are regulated by PACAP regardless when the peptide is administered. Few genes such as Homer 1, not affected by PACAP at early times of treatment can be regulated when the peptide is administered in a delayed manner and vice versa, few genes such as Hmox1 or Gjd2 are only regulated by PACAP when it is administered to the animals just after the reperfusion.

**Table 1:** Effect of acute and delayed IN PACAP administration after reperfusion (tMCAO protocol) on gene expression measured 48 h after the occlusion.

Events	Gene	ctrl vs sham			10 min vs ctrl			1 h vs ctrl			6 h vs ctrl			15 h vs ctrl		
			SEM	stat		SEM	stat		SEM	stat		SEM	stat		SEM	stat
Excitotoxicity	GJD2	**↘ 0.22**	**0.09**	***	**↗7.88**	**0.19**	****	↗1.83	0.1		↗1.01	0.05		↗1.66	0.12	
	GJB6	**↗1.29**	**0.06**	**	**↘0.34**	**0.07**	****	**↘0.72**	**0.12**	***	↗1.45	0.34		↗1.26	0.14	
	GAD1	↘0.88	0.19		↘0.378	0.44		**↗2.49**	**0.21**	**	**↗2.13**	**0.14**	*	**↗2.52**	**0.28**	**
	
Stress Response	CAT	↗1.56	0.38		**↘0.80**	**0.03**	***	**↘0.30**	**0.13**	**	↗1.04	0.14		**↘0.40**	**0.09**	**
	GPX1	**↗1.78**	**0.34**	*	↘0.58	0.4		↘0.10	0.32		↗1.157	0.17		↘0.61	0.07	
	NOS1	**↗2.26**	**0.18**	**	**↘0.46**	**0.21**	**	**↘0.50**	**0.14**	**	**↘0.54**	**0.22**	**	**↘0.58**	**0.19**	*
	HMOX1	**↗19.55**	**1.25**	***	**↘0.47**	**0.49**	**	↘0.84	3.57		↘0.87	1.52		↗1.02	2.97	
	JUN	**↗1.53**	**0.26**	*	↘0.83	0.19		↘0.30	0.14		↘0.87	0.31		**↘0.53**	**0.06**	**
	HSPA1B	**↗3.20**	**0.43**	*	↘0.40	0.53		↘0.42	0.1		↘0.92	0.58		↘0.61	0.38	
	HSPB1	**↗4.42**	**0.64**	**	↘0.77	0.93		↗1.04	0.45		↗1.09	0.44		↘0.72	0.58	
	HSPD1	**↗2.04**	**0.06**	*	**↘0.73**	**0.14**	*	↘0.59	0.16		↘0.81	0.31		↘0.87	0.27	
						
Apoptosis	DDIT3	↘0.78	0.23		↗1.05	0.23		↗1.11	0.16		**↗2.18**	**0.09**	**	↘0.844	0.25	
	DUSP6	↘0.75	0.26		↗1.73	0.41		**↗2.56**	**0.36**	*	**↗2.72**	**0.34**	*	↗1.66	0.2	
	BCL10	**↗1.90**	**0.05**	*	↘0.65	0.31		↘0.993	0.12		**↘0.17**	**0.03**	***	↘0.81	0.22	
	FAS	↗1.31	0.18		**↗2.89**	**0.21**	*	**↗3.39**	**0.65**	**	↗2.34	0.26		↗1.80	0.27	
	CASP9	**↗2.21**	**0.08**	****	**↘0.34**	**0.02**	****	**↘0.50**	**0.09**	***	**↘0.10**	**0.04**	****	**↘0.49**	**0.2**	***
	GADD45A	↘0.63	0.42		**↗2.34**	**0.19**	**	**↗2.90**	**0.24**	***	↘0.90	0.04		**↗1.97**	**0.15**	*
	ATF3	**↗37.34**	**2.24**	**	**↘0.16**	**0.07**	*	**↗2.64**	**4.2**	***	↗1.04	8.65		↗1.16	2.82	
	PIK3R1	↗1.05	0.11		↘0.78	0.27		**↘0.36**	**0.07**	**	↗1.10	0.13		↘0.65	0.09	
						
Inflammation	IL12A	↗2.20	0.64		↘0.49	0.55		↘0.92	0.41		**↘0.24**	**0.14**	*	↘0.84	0.2	
	IL6	**↗4.68**	**0.1**	****	**↘0.23**	**0.05**	****	**↘0.445**	**0.24**	****	**↘0.16**	**0.15**	****	**↘0.33**	**0.52**	****
	IFNAR1	**↗3.87**	**0.34**	*	**↘0.12**	**0.06**	*	↘0.63	0.32		**↘0.27**	**0.13**	*	↘0.48	0.85	
	IRF1	**↗2.74**	**0.52**	**	**↘0.49**	**0.17**	*	**↘0.47**	**0.18**	**	↗1.11	0.26		**↘0.43**	**0.21**	**
	IRF5A	**↗3.66**	**0.94**	*	**↘0.51**	**0.07**	*	↘0.73	0.98		↗1.48	0.58		↘0.44	0.27	
	CXCR4	**↗4.47**	**0.47**	***	**↘0.44**	**0.50**	*	**↘0.458**	**0.37**	*	↗1.09	0.57		**↘0.46**	**0.53**	*
	CXCR1	↗1.03	0.08		↘0.95	0.02		↗1.59	0.24		**↗1.99**	**0.44**	*	↗1.47	0.14	
	VCAM1	↘0.60	0.57		↗1.71	0.48		**↗1.47**	**0.2**	*	↗1.51	0.08		**↗1.96**	**0.13**	**
	TLR4	**↗4.06**	**0.33**	****	**↘0.58**	**0.28**	**	**↘0.78**	**0.03**	*	↘0.85	0.39		**↘0.52**	**0.23**	****
						
Synaptic/neurogenesis/Angiogenesis/cellular protection and activity	NEUROD1	↘0.48	0.55		**↗2.07**	**0.32**	**	↗1.08	0.1		**↗2.28**	**0.1**	*	↗1.32	0.04	
	BDNF	↘0.52	0.39		↗1.33	0.12		↗6.07	0.95		**↗3.31**	**0.21**	**	↗1.35	0.18	
	SLC16A7	↘0.74	0.18		↘0.20	0.03		↘0.60	0.16		↘0.99	0.21		**↗1.91**	**0.18**	*
	DBI	**↘0.37**	**0.06**	**	**↗2.83**	**0.46**	*	↘0.99	0.1		↗1.26	0.09		↗1.53	0.14	
	VEGFA	**↘0.47**	**0.15**	**	**↗1.75**	**0.27**	**	↗1.19	0.1		**↗2.32**	**0.08**	***	↘0.93	0.07	
	TGFB2	↗1.02	0.15		↗1.55	0.26		↗1.17	0.08		↗1.27	0.11		**↗1.76**	**0.33**	*
	TGFB1	**↗6.46**	**0.66**	****	**↘0.40**	**0.06**	****	**↘0.49**	**0.95**	**	**↘0.54**	**0.47**	***	**↘0.16**	**0.32**	****
	TGFBR1	↗1.25	0.17		↗1.73	0.52		↗1.44	0.08		↗1.30	0.18		↘0.83	0.24	
	GFAP	**↗21.61**	**3.71**	***	**↘0.42**	**1.03**	**	↘0.80	3.13		↘0.86	1.88		**↘0.52**	**3.46**	*
	HOMER1	**↘0.27**	**0.53**	*	↘0.17	0.09		↗1.72	0.12		**↗3.59**	**0.25**	*	↗1.62	0.16	
	TIMP1	**↗46.90**	**8.63**	***	↘0.75	0.59		↘0.74	2.56		**↘0.36**	**1.85**	**	**↘0.456**	**4.55**	*
	TPA	**↗7.95**	**0.57**	****	**↘0.17**	**0.03**	****	**↘0.52**	**1.32**	**	**↘0.17**	**0.21**	***	**↘0.334**	**1.04**	***
	AQP4	**↗2.86**	**0.22**	**	**↘0.61**	**0.23**	*	↘0.59	0.19		**↘0.43**	**0.27**	*	↘0.95	0.61	
	ACVR1	**↗2.33**	**0.22**	**	**↘0.34**	**0.08**	**	**↘0.36**	**0.23**	**	**↘0.28**	**0.17**	**	↘0.36	0.33	

PACAP was administered 10 min, 1 h, 6 h, or 15 h after reperfusion. Ctrl corresponds to animal subjected to tMCAO and sham corresponds to operated animals who were not subjected to tMCAO. Results are expressed in relation to the ones of sham ± SEM. (*P < 0.05; **P < 0.01; ***P < 0.001; ****P < 0.0001). The arrow also indicate a up and down gene regulation.

### PACAP Dose Response Efficiency

**Figure 2:**
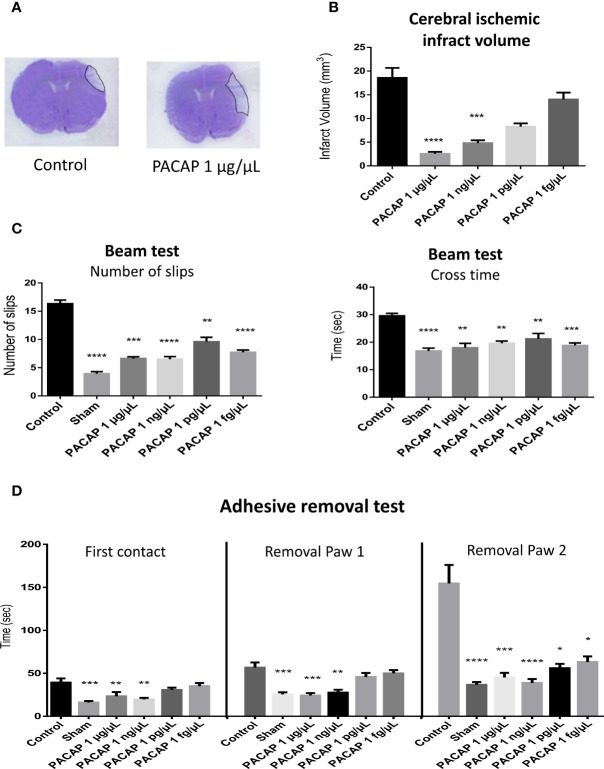
Effects on the infarct volume and functional recovery, 2 days after stroke, of a single intranasal (IN) administration of decreasing concentrations of PACAP provided 1 h after pMCAO. Male C57BL6 mice were treated with a saline solution (NaCl 0.9% for control and sham) or with 10 µl of different PACAP concentrations (1 µg/µl, 1 ng/µl, 1 pg/µl, and 1 fg/µl dissolved in NaCl 0.9%). **(A)** Illustration of the brain infarct zone after NaCl (Control) or PACAP 1 µg/µl IN administration, 48 h after pMCAO. **(C)** Cerebral ischemic infarct volume (mm^3^) 48 h after pMCAO. **(C)** Beam test indicating cross times and number of slips of mice on 9-mm beam. **(D)** Adhesive removal test with the time taken for first contact and adhesive removal from mice paws 2 days after stroke. PACAP treatment promoted functional recovery and reduced cerebral ischemic lesions in stroke mice. Variations of n ≥ 6 animals per group are reported as S.E.M. (*P < 0.05; **P < 0.01; ***P < 0.001; ****P < 0.0001).

After putting in evidence the efficiency of an IN administration of PACAP for stroke treatment and the existence of a therapeutic window of a least 6 h in the tMCAO mice model, we looked for the most efficient dose of the peptide. For those experiments, the neuroprotective effect of a single IN administration of different concentrations of PACAP ranging from 1 µg/µl to 1 fg/µl, 1 h after permanent middle cerebral artery occlusion (pMCAO) was tested. The choice of using a pMCAO mice model was both to evaluate the efficiency of the treatment in another commonly used stroke model as recommended by the STAIR guidelines and to reduce the number of animals used, given that reproducibility and survival rate in the pMCAO is higher than in the tMCAO. Effectively we recorded that the mortality rate in tMCAO model (20%) was approximately twice higher than in the pMCAO one. The other advantage of the pMCAO model is that it produces a smaller infarct area more in line with what is observed in human stroke (42). Results highlight that a single IN administration of very low concentrations of PACAP decreased infarct volume (**Figures 2A, B**) when administered 1 h after stroke in a pMCAO mice model. Effectively, 48 h after the occlusion, an 86.3% reduction of the infarct volume compared to controls was observed when animals received 1 µg/µl IN PACAP treatment (**Figures 2A, B**). This protection diminished when decreasing the doses of PACAP, with a reduction of the infarct volume of only 24.6% for animals receiving 1 fg/µl of PACAP (**Figure 2B**). In addition, to these histological effects, PACAP also improved functional recovery (**Figures 2C, D**). In particular, PACAP administered 1 h after pMCAO decreased the number of slips from 16.3 ± 0.6 for control mice to 6.6 ± 0.3 for animals treated with the highest concentration of PACAP (**Figure 2C**). PACAP also reduced the time spent to cross the beam from 29.6 ± 0.9 s for control mice to 18 ± 1.6 s for animals treated with 1 µg/µl PACAP. Finally, PACAP treatment enhanced performances in the adhesive removal test compared to control animals after pMCAO with asymmetrical symptoms (**Figure 2D**). It is interesting to note that several of those tests revealed functional improvement even with the lowest doses of PACAP (**Figures 2C, D**).

### PACAP Delayed IN Daily Administration Efficiency

**Figure 3:**
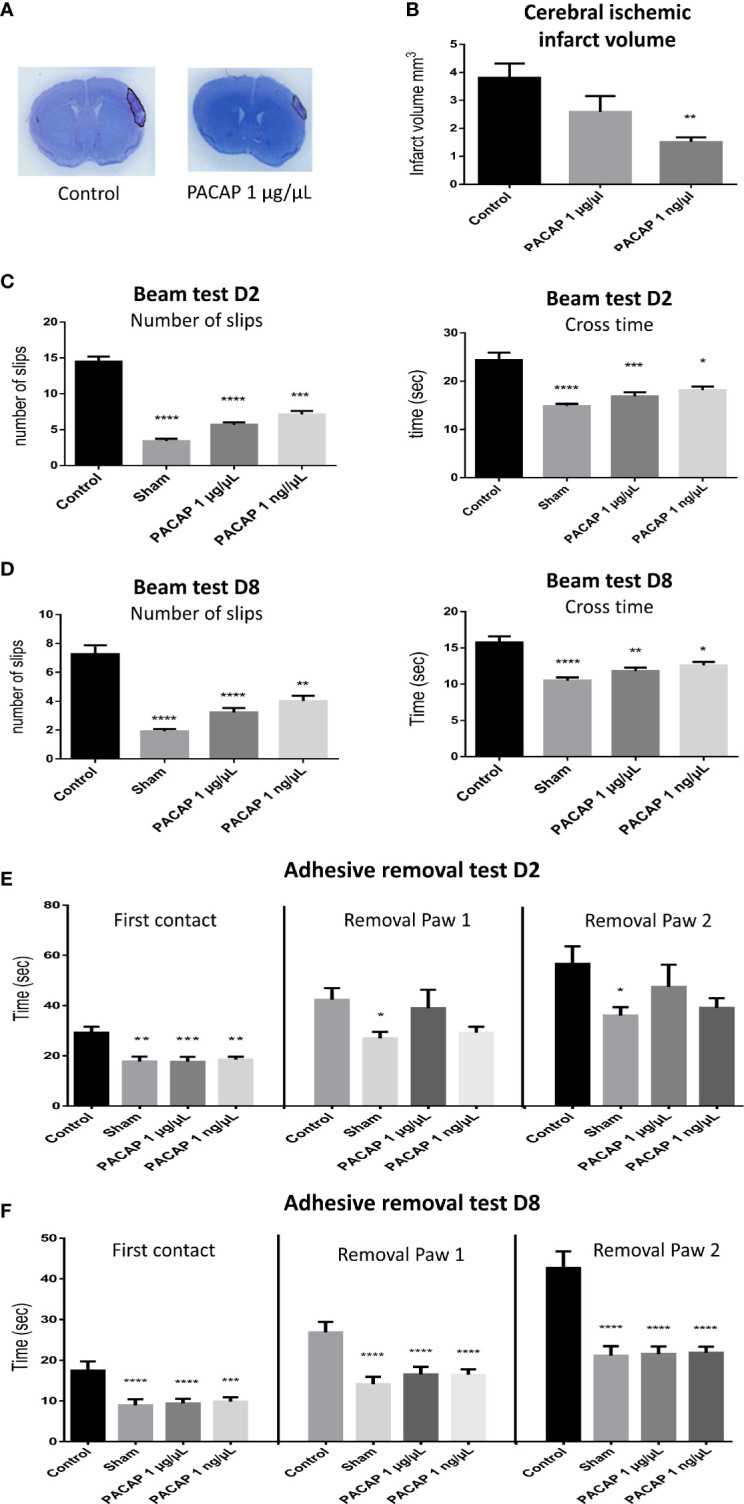
Effects on the infarct volume and functional recovery of delayed (6 h post pMCAO) PACAP daily administration, 2 and 8 days after brain ischemia. Male C57BL6 mice were treated with a saline solution (control, sham) or with different concentrations of PACAP. **(A)** Illustration of the brain infarct zone after NaCl (control) or PACAP 1 µg/µl intranasal administration, 8 days after pMCAO. **(B)** Cerebral ischemic infarct volume (mm^3^) 8 days after pMCAO. **(C, D)** Beam test indicating cross times and number of slips of mice (sham and stroked animals) on 9 mm beam 2 and 8 days after stroke. **(E, F)** Adhesive removal test with the time taken for first contact and adhesive removal from mice paws 2 and 8 days after stroke. PACAP treatment promoted functional recovery and reduced cerebral ischemic lesions in stroke mice. Errors are reported as Mean ± S.E.M and n ≥ 8 animals per group. (*P < 0.05; **P < 0.01; ***P < 0.001, ****P < 0.0001).

The efficacy of an IN administration of PACAP at very low doses in a pMCAO mice model being demonstrated, we tested whether the treatment is still efficient and persistent when started 6 h after ischemia in the pMCAO mice model. For those experiments, animals received 1 µg/µl or 1 ng/µl daily administration of PACAP. The results show that at day 8, the infarct volume of 3.8 ± 0.5 mm^3^ for mice from the control group was reduced to 2.6 ± 0.6 and 1.5 ± 0.2 mm^3^ for animals treated intranasally with 1 µg/µl and 1 ng/µl of PACAP, respectively (**Figures 3A, B**). Such treatments also improved the sensorimotor performances by reducing on the beam test, the number of slips and the time spent to cross the beam both 2 and 8 days after the ischemia (**Figures 3C, D**) and by reducing for the adhesive removal test, the time spent to feel the presence of the adhesive and to withdraw it from both forepaws (**Figures 3E, F**). In most pMCAO mice, an asymmetrical symptom was observed with firstly withdrawal of the adhesive from the right paw, which is controlled by the ipsilateral hemisphere, before elimination in a second time of the adhesive from the left paw, which is controlled by the contralateral hemisphere.

### Food Intake and Body Weight Variation

**Figure 4:**
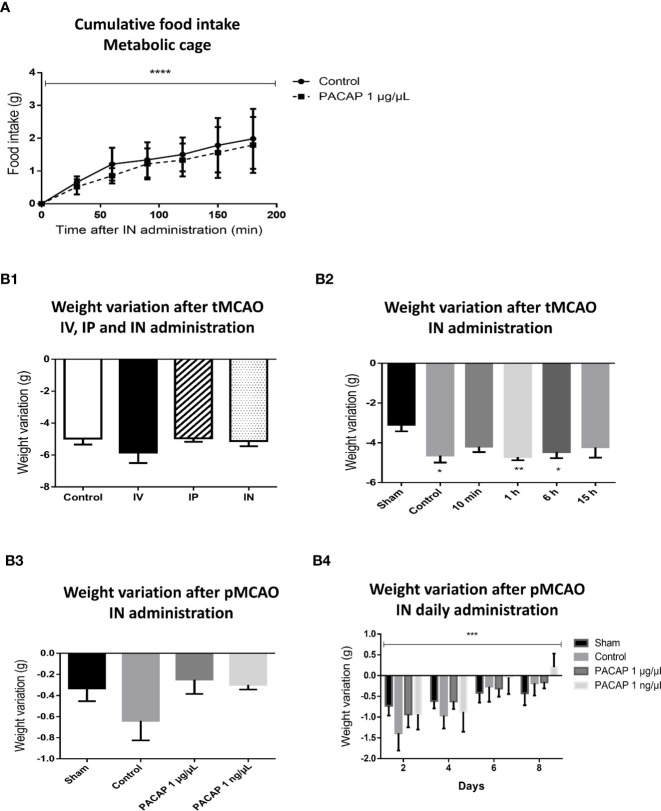
Effects of stroke and/or PACAP treatment on cumulative food intake and body weight variation. **(A)** Cumulative food intake after PACAP IN delivery over 3 h of time in non-operated mice. **(B)** Variation of body weight after PACAP administration following different stroke protocols, i.e., PACAP administration routes protocol (B1.), PACAP therapeutic window protocol (B2.), PACAP dose response efficiency protocol (B3.) and PACAP delayed IN daily administration efficiency protocol (B4.). Errors are reported as Mean ± S.E.M. (*P < 0.05; **P < 0.01; ***P < 0.001). Two-way ANOVA was performed for figure A and B4 showing significant means only for time factor (minutes and days), while the effects of treatment and the interaction are not significant.

The anorexigenic effects of PACAP have mainly been studied in various species such as chickens (43), fishes (44, 45), and rodents (46, 47), using different routes of administration from systemic ones such as IV or IP to direct brain administrations such as ICV or amygdala central nucleus injections. In the present study the effect of IN administration of PACAP was evaluated on body weight variation and food intake. The results showed no significant differences between control and PACAP treated groups in terms of food intake (**Figure 4A**). Interestingly, weight loss was usually slightly less important after PACAP IN acute or delayed administration, 2 and 4 days after pMCAO (**Figure 4B**). A significant decrease in the weight loss was even observed in the pMCAO model after 8 days of 1 ng/µl PACAP daily IN administration. Those experiments also highlight that the weight loss is significantly more important for the mice subjected to a tMCAO than for the animals included in the pMCAO protocol, which is probably due to the heavier surgery that represents the intraluminal filament technique, comforting the necessity to use various stroke models which have different outcomes that may interfere differently with the subsequent treatment.

## Discussion

Stroke is the second leading cause of death worldwide and the leading cause of disability in adults. The complex processes involved in stroke induced brain damages explain the difficulties to develop effective pharmacological agents, making it a major health problem. Thus so far, reperfusion is the only approach really recommended but its use is restricted because of cost, short therapeutic window or risks of causing a hemorrhage, so that it can only benefit to a small proportion of stroke victims. This therefore led over the last couple of years to intense research targeting some of the numerous mechanisms activated after brain artery occlusion such as excitotoxicity, oxidative stress, inflammation and apoptosis in order to block neuronal cell death and/or stimulate neurogenesis, synaptogenesis and angiogenesis in order to promote functional recovery. However, most trials so far have failed, probably because molecules could only target part of stroke induced deleterious pathways. In this context, PACAP may be a promising molecule as it could counteract most if not all the deleterious processes induced by stroke and improve functional recovery in various MCAO models (39–48). However, to consider the use of PACAP in clinic, several points remain to be evaluated and/or improved. Indeed, PACAP, acting *via* 3 different receptors (PAC1, VPAC1, and VPAC2) widely distributed in the brain and peripheral organs, regulates numerous biological functions, including hormone release or systemic decrease of blood pressure. It seems to be the VPAC2 receptor, highly expressed in the cardiovascular system and endocrine glands that is at the origin of most of the deleterious side effects of PACAP. Conversely, VPAC2 expression is very low in the brain (14).

In addition, although PACAP remains active after IV administration by passing the blood-brain barrier (BBB), it is very quickly degraded in blood with a half-life of less than 5 min due to a high sensibility to dipeptidyl-peptidase-IV (DPP-IV), endopeptidases, and carboxypeptidases. In particular, PACAP is hydrolyzed into PACAP3-38 acid amins and PACAP5-38 to create a shorter peptide with antagonist activity which may limit the effect of the treatment (34, 49, 50).

### PACAP Administration Routes

Various administration routes of PACAP have already been reported from IP to intravitreal administration (16–51), leading either to systemic or topical delivery of the peptide. This diversity of PACAP administration routes led us to compare the efficiency of a single intranal (IN), IV, and IP administration of the peptide after tMCAO in C57Bl/6 mice. The results revealed that the IN PACAP delivery led to the stronger neuroprotection, which could be explained by the fact that the quantity of PACAP able to reach the brain is variable, depending on i) the amount provided and ii) the route of administration. It is also likely that IN administration induces a direct delivery of PACAP to the brain through a rapid absorption *via* the nasal mucosa with highest uptake of the peptide in the occipital cortex and striatum (52). At the same time, IN delivery reduces systemic exposure, decreasing by the way peripheral side effects, in particular on the cardiovascular system.

### Food Intake and Weight Variation

It is well established that PACAP regulates food intake and energy homeostasis through both central and peripheral action. In particular, PACAP injected centrally into the hypothalamic ventromedial nucleus or into the posterior region of the stria terminalis bed nucleus induced a rat weight loss 24 h post injection (53, 54). Also, an IP administration of PACAP to PAC1-deficient and wild-type mice shows that PACAP suppresses appetite *via* its PAC1 receptor by inhibiting ghrelin and increasing GLP-1 and leptin (55). The anorexigenic effect was also observed after IV and ICV administration of high concentrations of PACAP (56, 57). Although our results seem to be in contradiction with these previous reports, the discrepancy might be due to differences in the amount of PACAP which reaches the various brain regions after IN administration. It is estimated that less than 4% of the 10 µg of PACAP administered to the mice actually reach the brain (52). Furthermore, the main brain area playing a role in appetite regulation and energy homeostasis are hypothalamus and amygdala (58, 59) while the IN administration tend to promote peptide uptake toward the occipital cortex and striatum (52). In addition to those experiments on food intake, further thorough tests must be done to confirm the innocuity of this efficient, non-invasive and easy to set up IN administration of PACAP (60, 61). Moreover, this approach has started to be used with PACAP for the treatment, in animal models, of some neurodegenerative diseases such as Alzheimer or Huntington (23, 27, 62).

### PACAP Therapeutic Window

The present results demonstrate that the therapeutic window of PACAP in mice could exceed 6 h. This observation is very promising when considering that rtPA, the approved medical treatment for acute ischemic stroke, has a therapeutic window of only 2 h in mice (63). Considering that in clinic rtPA therapeutic window reaches 4.5 h, we can extrapolate that in human, PACAP could still block development of the infarct area when injected 12 h after the ischemic event.

In mice, 15 h after reperfusion the infarct area is largely consolidated (64), so it is not surprising that PACAP has no more ability to reduce the lesion volume. Nevertheless, the gene expression data suggest that a delayed administration of PACAP after stroke could still shift the inflammatory response and stimulate synaptogenesis. These data correlate with the observation that injection of PACAP-producing stem cells, 3 days after permanent focal ischemia, do not reduce the ischemic lesion volume but shift the inflammatory response from a M1 to M2 phenotype and promote functional recovery (31).

### PACAP Dose Response Efficiency

In accordance with the tMCAO protocol observations, a single IN administration of a very low dose of PACAP in a pMCAO also decreased infarct volume and enhanced sensorimotor performances, in accordance with what was previously reported through other routes of administration [(65, 66) and others referenced higher]. The fact that low doses of PACAP are sufficient to protect the brain from stroke, together with publications showing that PACAP-deficient mice exhibit increased lesions and enhanced neurological deficits (39, 67–69) suggests that any molecule, such as linagliptin, susceptible to increase endogenous PACAP levels could be of interest for the treatment of stroke.

### PACAP Delayed IN Daily Administration Efficiency

Administration of PACAP started 6 h after pMCAO and repeated daily for 6 days led to a significant improvement in neurological function recovery and a reduction of the infarct volume. Surprisingly, when PACAP was administered 6 h after ischemia, the most important infarct volume reduction was observed in mice treated with the lowest concentration of PACAP (1 ng/µl/day). These results suggest that a daily reiteration of PACAP administration could at high doses induce desensitization of PAC1 receptors through sequestration away from the membrane (70). Nevertheless, the PACAP treatment remained efficient and this result highlights the potential of low doses of PACAP, which will certainly decrease the risk of side effects.

### Gene Expression

Ischemia is known to cause an early wave of glutamatergic excitotoxicity on depolarizing or dying neurons, resulting in breakdown of the homeostatic and water balances. Interestingly, several genes such as GAD1, GJB6, GJD2, and AQP4 known to be induced or repressed by those early events of stroke (71–74) are regulated in an opposite manner by PACAP which contributes to explain how PACAP protects the brain after stroke. For instance, PACAP by inhibiting AQP4, reduces brain oedema, and promotes cell survival after MCAO (75, 76). Likewise, PACAP injections 10 min or 1 h after reperfusion probably inhibit the deleterious action of glutamate by down-regulating GJB6 and up-regulating GJD2 (77). The observed repression of Tpa mRNA expression should also reduce stroke induced NMDA receptor-mediated signaling (78). Conversely, the fact that GAD1 expression is increased when PACAP is injected 1, 6, and 15 h after reperfusion suggests that PACAP also contributes to restore altered GABA levels after stroke (79), which must in turn reduce neuronal hyperexcitability.

Homeostatic disruption and the massive entry of ions inside the cell provoke a reactive oxygen/nitrogen species overproduction such as nNOS and iNOS (80, 81) causing severe intracellular damages to macromolecules and mitochondrial impairment, leading to apoptosis. It is thus interesting to note that PACAP represses the ischemic induction of NOS1 (a gene encoding nNOS) mRNA expression, which attenuates free radical production and in turn reduces oxidative stress. This may explain that 48 h after reperfusion, the expression of genes from the antioxidant system such as CAT, GPX1 and HMOX1 is reduced in PACAP treated animals. In addition, PACAP induction of GADD45A and GADD153/DDIT3 mRNA expression contributes to repair oxydative stress induced DNA damage (82–84), which reduces the number of cells entering apoptosis. The ability of PACAP to repress the expression of pro-apoptotic genes such as Caspase 9, PIK3R1, ATF3, FAS, or BCL10 and to promote the expression of anti-apoptotic genes such as Dusp6 provides further information on how PACAP prevents neuronal cell death. Of course, other molecular players are also involved in this neuroprotection mechanism. For example the blockage by PACAP of stroke induced c-Jun mRNA expression, supports the idea that it blocks apoptosis through an inhibition of the expression of proteins such as bax and a concomitant stimulation of BCL2 or BCL-XL as already shown in other experimental models (29, 85, 86). Besides its neuroprotective effect, PACAP also reduces reactive astrogliosis as shown by its ability to inhibit stroke induced GFAP and VIM expression. Such effect of PACAP on GFAP and VIM expression decreases post-stroke astrocytic hypertrophy and contributes to brain protection (87–89).

Within minutes to hours after ischemia, an inflammatory response is initiated (41–90) whose intensity is strongly correlated with stroke severity (91). As already shown PACAP can skew the inflammatory response from a deleterious phenotype to a protective one (28, 31, 67). The present results obtained with TLR4, IL6, or IRF1 confirm that PACAP can inhibit most proinflammatory mediators both when given rapidly or in a delayed manner after ischemia.

The ability of PACAP to stimulate the expression of genes such as BDNF, SLC16A7, NEUROD1, VEGFA, HOMER 1, or DBI/ACBP when administered either just after the occlusion or at later time points, highlights the ability of PACAP to not only block stroke induced apoptosis but also to promote subsequent synaptic plasticity and brain regeneration.

Taken together, the present gene expression results highlight the many protective pathways activated by PACAP after stroke both when administered within minutes after the reperfusion but still when provided 15 h after the stroke onset. One of the strengths of PACAP is probably its ability to reduce both neuronal cell death in the acute phase of stroke, to sustainably skew the inflammatory response toward a protective phenotype and to promote during the chronic phase of stroke the release of neurotrophic factors responsible for subsequent neuronal plasticity.

## Conclusion and Future Perspectives

Neuronal cell death triggered by MCAO is initiated by several cellular events including excitotoxicity, free radical damages and inflammation which often activate apoptosis. Current research is focusing on candidate-drugs that may slow or prevent brain ischemic injury. There have been many unsuccessful therapeutic trials in stroke due to a single angle of attack. PACAP by modulating simultaneously different deleterious pathways activated by stroke could be a promising therapeutic molecule for the treatment of brain ischemia. However, the rapid degradation of the peptide and its numerous effects on the body require a method of delivery which targets specifically the brain. In this perspective, the present results showing the high efficiency of an IN PACAP administration are very promising. Future development should focus on the use of PACAP analogs and vectorization methods to increase the half-life of the peptide and its selectivity for PAC1 and VPAC1 receptors to further enhance its neuroprotective action while reducing possible side effects. It will also be interesting to study a potential combination therapy with PACAP and traditional stroke reperfusion methods (rtPA or endovascular thrombectomy) in order to see if PACAP can act as a freezing molecule of the penumbra to enhance the therapeutic window of the already approved reperfusion methods (6).

## Data Availability Statement

The raw data supporting the conclusions of this article will be made available by the authors, without undue reservation.

## Ethics Statement

The animal study was reviewed and approved by the Regional committee of ethics for animal experimentation (CENOMEXA; approval number #7619-2016101417048165) and conducted in an accredited animal facility (C7645104), according to the recommendations of the European Union under the supervision of authorized investigators.

## Author Contributions

AC and JM performed the experiments. BL and JL synthesized PACAP. Supervision and conceptualization were performed by DV. AC and DV wrote the manuscript. AC, JM, BL, JL, and DV revised the manuscripts. Final editing was performed by AC and DV. All authors contributed to the article and approved the submitted version.

## Funding

AC was awarded a postdoctoral fellowship in neuroscience provided by Battuta Erasmus Mundus with the support of the European Commission and the cooperation of Badji Mokhtar University-Annaba, Algeria. This work was supported by INSERM (U1239), Rouen University, Normandy Region and the European Union. Europe gets involved in Normandy with European Regional Development Fund (ERDF).

## Conflict of Interest

The authors declare that the research was conducted in the absence of any commercial or financial relationships that could be construed as a potential conflict of interest.

## Abbreviations

ACVR1, Activin A receptor 1; AQP4, Aquaporin 4; ATF3, Activating transcription factor 3; BBB, Blood brain barrier; BCL10, B cell lymphoma/leukemia 10; BDNF, Brain derived neurotrophic factor; CASP9, Caspase 9; CAT, Catalase; CBF, Cerebral blood flow; CXCR1, Chemokine receptor type 1; CXCR4, Chemokine receptor type 4; DBI, Diazepam binding inhibitor; DDIT3, DNA damage inducible transcript 3; DUSP6, Dual specificity phosphatase 6; FAS, Fas cell surface death receptor; GAD1, Glutamate decarboxylase 1; GADD45A, Growth arrest and DNA damage inducible alpha; GFAP, Glial fibrillary acidic protein; GJB6, Gap junction beta 6; GJD2, Gap Junction Protein Delta 2; GPX1, Glutathione peroxidase 1; HMOX1, Heme oxygenase 1; HOMER1, Homer scaffold protein 1; HSPA1B, Heat shock protein A (Hsp70) 1B; HSPB1, Heat shock protein beta 1; HSPD1, Heat shock protein D (Hsp60) 1; IFNAR1, Interferon alpha and beta receptor subunit 1; IL12A, Interleukin-12 subunit alpha; IL6, Interleukin-6; IN, Intranasal Injection; IP, Intraperitoneal injection; IRF1, Interferon regulatory factor 1; IRF5A, Interferon regulatory factor 5a; IV, Intravenous injection; JUN, Jun proto-oncogene; MCAO, Middle cerebral artery occlusion; NEUROD1, Neurogenic differentiation 1; NOS1, Nitric oxide synthase 1; PAC1, Pituitary adenylate cyclase-activating polypeptide type I receptor; PACAP, Pituitary adenylate cyclase-activating polypeptide; PIK3R1, Phosphatidylinositol 3-kinase regulatory subunit alpha; pMCAO, permanent middle cerebral artery occlusion; SLC16A7, Solute Carrier Family 16 Member 7 (Monocarboxylate Transporter 2) TGFB1, Transforming growth factor-beta 2; TGFB2, Transforming growth factor-beta 2; TGFBR1, Transforming growth factor-beta receptor type 1; TIMP1, TIMP metallopeptidase inhibitor 1; TLR4, Toll-like receptor 4; tMCAO, Transient middle cerebral artery occlusion; tPA, Tissue plasminogen activator; VCAM1, Vascular cell adhesion molecule 1; VEGFA, Vascular endothelial growth factor A; VIP, Vasoactive intestinal polypeptide; VPAC1, Vasoactive intestinal polypeptide receptor 1; VPAC2, Vasoactive intestinal polypeptide receptor 1.
